# Supplementary material for: Urinary vesicle biomarkers and kidney function—results from the German AugUR study
Source: Sci Rep. 2026 Jun 4;16:17350. doi: 10.1038/s41598-026-56331-w (PMC13237031; doi:10.1038/s41598-026-56331-w)
Supplement: Supplementary file 1 — Supplementary Material 1 [file 41598_2026_56331_MOESM1_ESM.docx]

# Supplementary information

Formula 1a. Normalization of vesicular albumin to suspension volume and volume of urine

$$\frac{vesicular albumin concentration\left( \frac{pg}{mL} \right)x EV suspension volume (mL)}{Volume of urine used (mL)}$$

Formula 1b. Normalization of vesicular podocalyxin to suspension volume and volume of urine

$$\frac{vesicular podocalyxin concentration \left( \frac{pg}{mL} \right)x EV suspension volume (mL)}{Volume of urine used (mL)}$$

Formula 2a. Normalization of vesicular albumin concentration on urinary creatinine concentration

$$vACR \left( \frac{ng}{g} \right)=\frac{normalized vesicular albumin concentration \left( \frac{ng}{L} \right)}{urinary creatinine \left( \frac{mg}{dL} \right)}*100$$

Formula 2b. Normalization of vesicular podocalyxin concentration on urinary creatinine concentration

$$vPCR \left( \frac{ng}{g} \right)=\frac{normalized vesicular podocalyxin concentration \left( \frac{ng}{L} \right)}{urinary creatinine \left( \frac{mg}{dL} \right)}*100$$

Formula 3a. Calculation of intra-assay coefficient of variation (CV)

$$CV=\frac{Standard deviation}{mean}$$

Formula 3b. Calculation of inter-assay coefficient of variation (CV)

$$CV=\frac{Standard deviation of plate means/values}{mean of plate means/values}$$

Vesicular albumin and podocalyxin concentrations were determined on two different days a week apart in triplicate (n = 26). Intra- and inter-assay CVs were calculated and the mean, as well as the minimum and maximum of the individual CVs is reported in Supplementary Table 1. After the initial single determination of vesicular albumin and podocalyxin concentrations lysed samples were stored for up to 17 months at -80°C before concentrations of the two markers were determined again as part of the triplicate measurements. Inter-assay CV was also calculated between the two triplicate measurements and the initial vesicular albumin and podocalyxin measurement.

**Supplementary Table 1.** Intra- and inter-assay CVs of triplicate measurements of vesicular albumin and podocalyxin.

|  | Vesicular albumin  (n = 26) | Vesicular podocalyxin  (n = 26) |
| --- | --- | --- |
| Intra-assay CV | 5.29% (0.06 – 16.86%)  9.76% (0.64 – 49.88%) | 5.53% (0.95 – 16.14%)  4.78% (1.60 – 13.85%) |
| Inter-assay CV (within one week) | 16.76% (0.51 – 137.62%) | 5.98% (0.57 – 23.29%) |
| Inter-assay CV (after 15 – 17 months) | 27.28% (8.57 – 166.77%) | 13.12% (2.96 – 37.13%) |

Mean (Min – Max) of individual CVs.

**Isolation of urinary vesicles and quantification of vesicular albumin and podocalyxin**

In the pilot study from one sample, 1403 vesicles were counted from which 269 were podocalyxin-positive. Based on this first estimation 19.1% of urinary vesicles seem to be derived from podocytes. Furthermore, these results lead to the assumption that podocalyxin is part of the vesicle membrane, as the antibody binds without prior permeabilization of the vesicle membrane (Supplementary Figure 1).


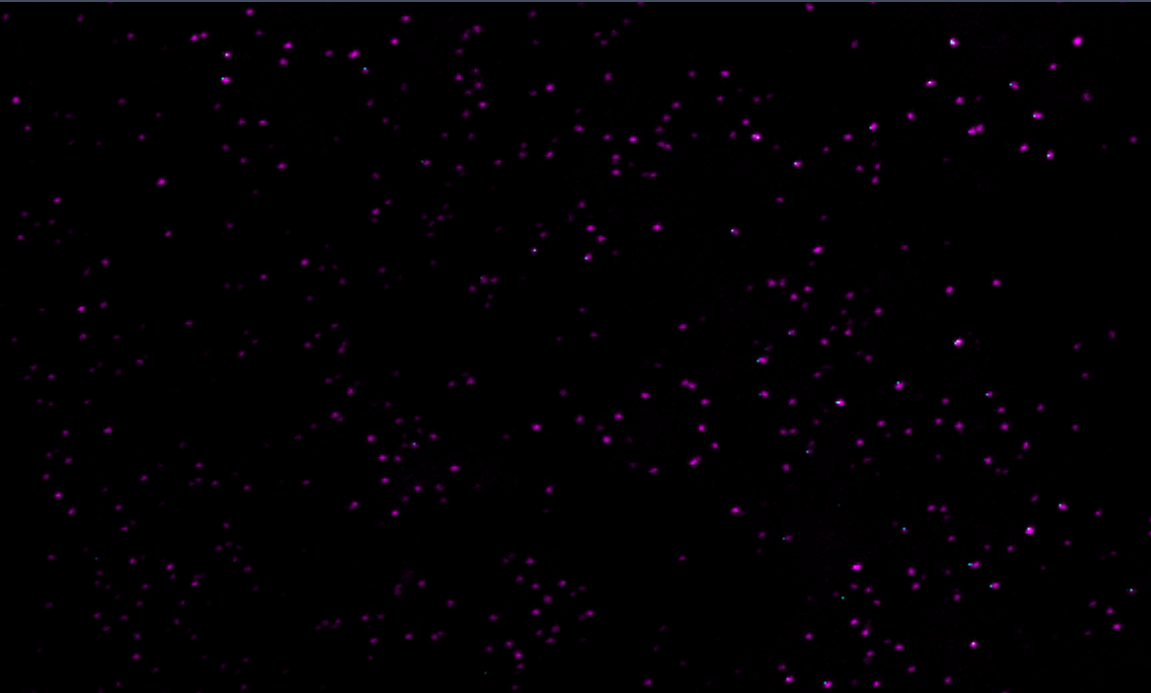


**Supplementary Figure 1.** Co-staining of the lipid bilayer of urinary extracellular vesicles with MemGlow^TM^ 560 (purple) and the podocyte-specific marker podocalyxin with the antibody PODXL488 (cyan).


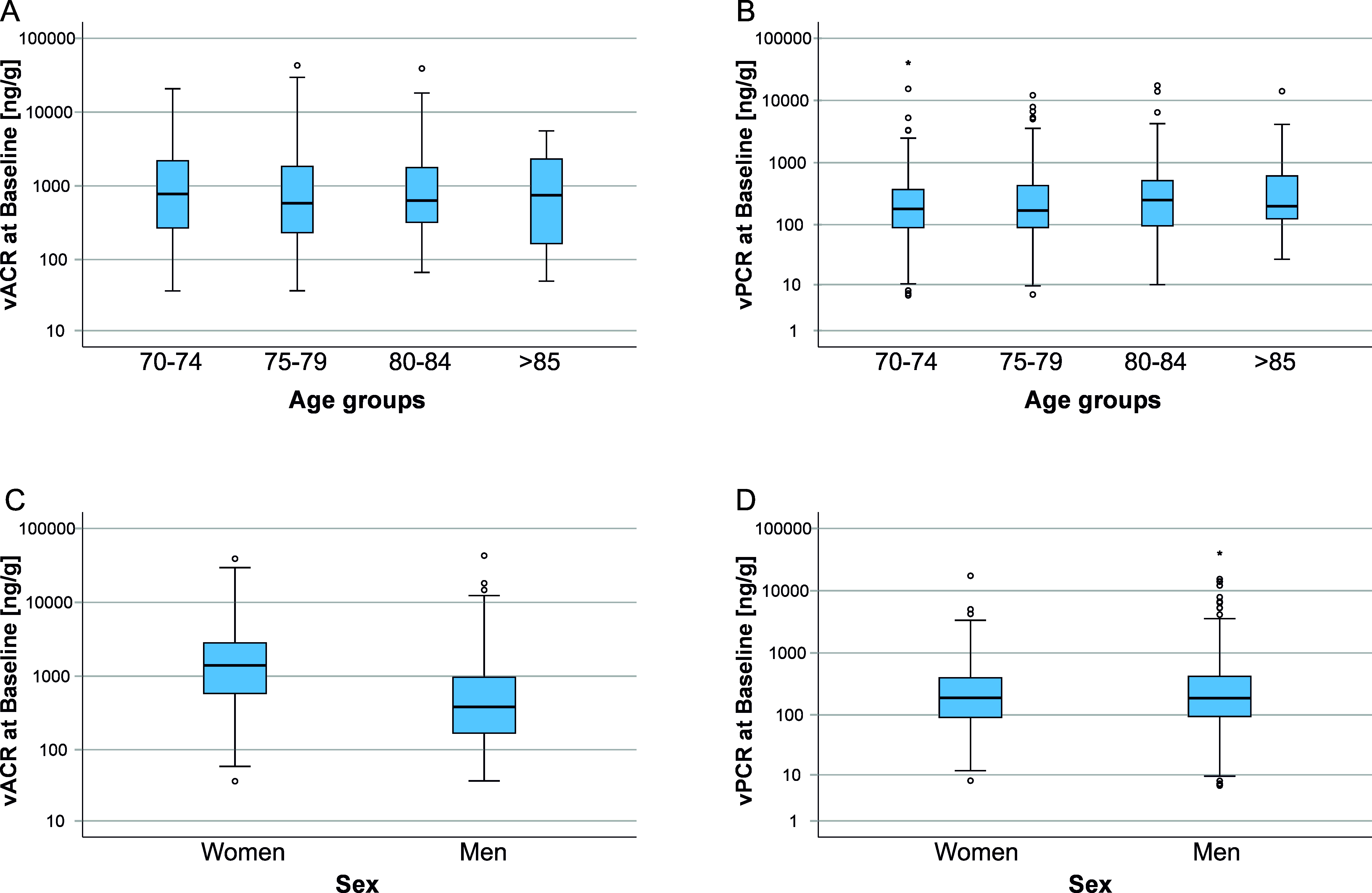


**Supplementary Figure 2.** Box plots visualising vACR (left) and vPCR (right) distributions (log-scale on y axis) at baseline over age groups (A, B) and by sex (C, D). vPCR significantly differed by age and vACR by sex.

To investigate whether any other factors might influence the association between sex and vACR, all variables from Table 1 were added to the linear regression model for vACR using a stepwise procedure. This revealed that the estimate of the effect of sex on vACR remained stable. It can therefore be concluded that none of the factors examined explain the association between sex and vACR.


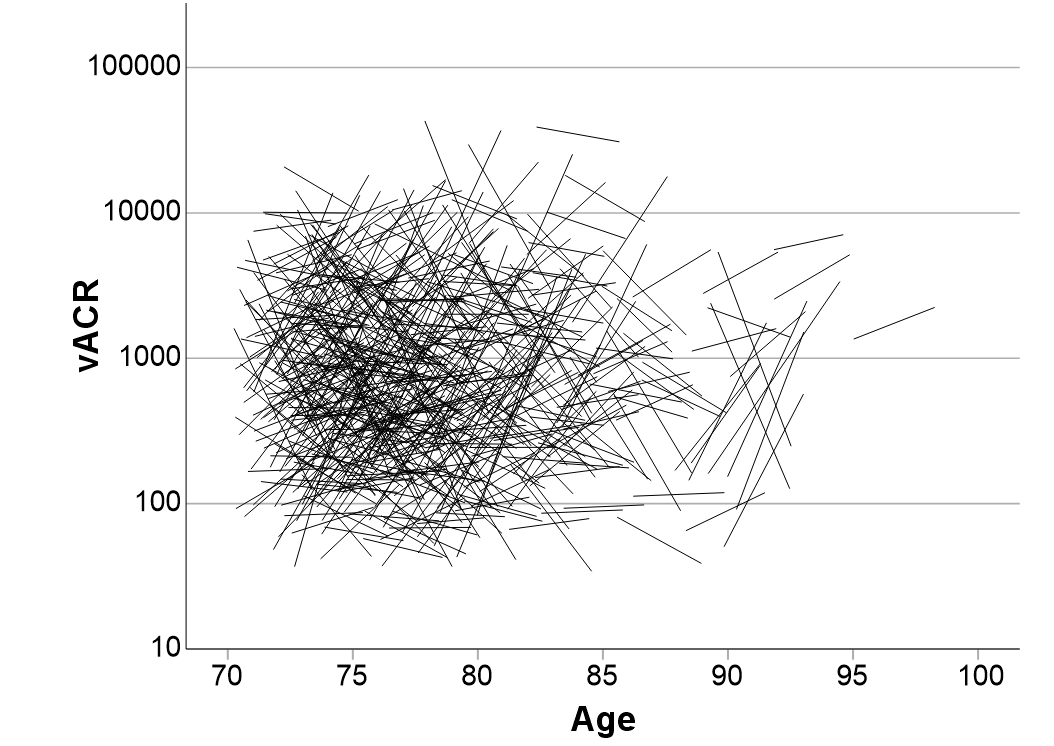


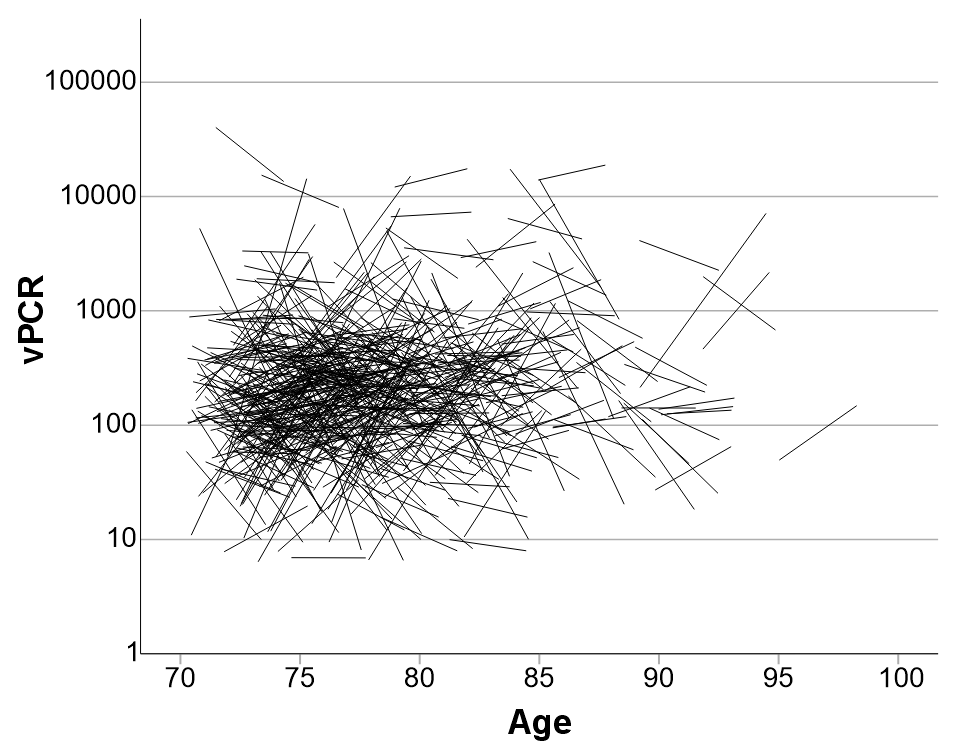


**Supplementary Figure 3.** Spaghetti plots for change in vACR (top) and vPCR (bottom) between baseline and three years follow-up over baseline age without normalization on baseline levels. A total of n=268 decreased and n=312 increased for vACR, as well as n=280 decreased and n=300 increased for vPCR.
